# Development of Antibacterial and Antifouling Innovative and Eco-Sustainable Sol–Gel Based Materials: From Marine Areas Protection to Healthcare Applications

**DOI:** 10.3390/gels8010026

**Published:** 2021-12-28

**Authors:** Ileana Ielo, Fausta Giacobello, Angela Castellano, Silvia Sfameni, Giulia Rando, Maria Rosaria Plutino

**Affiliations:** 1 Institute for the Study of Nanostructured Materials, ISMN—CNR, c/o Department of ChiBioFarAm, University of Messina, Viale F. Stagno d’Alcontres 31, Vill. S. Agata, 98166 Messina, Italy; ileana.ielo@ismn.cnr.it (I.I.); fausta.giacobello@ismn.cnr.it (F.G.); angela.castellano@ismn.cnr.it (A.C.); ssfameni@unime.it (S.S.); 2 Department of Engineering, University of Messina, Contrada di Dio, Vill. S. Agata, 98166 Messina, Italy; 3 Department of Chemical, Biological, Pharmaceutical and Analytical Sciences (ChiBioFarAm), University of Messina, Viale F. Stagno d’Alcontres 31, Vill. S. Agata, 98166 Messina, Italy; giulia.rando@unime.it

**Keywords:** antifouling coatings, sol–gel technique, antibacterial activity, marine protection, cultural heritage conservation

## Abstract

Bacterial colonization of surfaces is the leading cause of deterioration and contaminations. Fouling and bacterial settlement led to damaged coatings, allowing microorganisms to fracture and reach the inner section. Therefore, effective treatment of surface damaged material is helpful to detach bio-settlement from the surface and prevent deterioration. Moreover, surface coatings can withdraw biofouling and bacterial colonization due to inherent biomaterial characteristics, such as superhydrophobicity, avoiding bacterial resistance. Fouling was a past problem, yet its untargeted toxicity led to critical environmental concerns, and its use became forbidden. As a response, research shifted focus approaching a biocompatible alternative such as exciting developments in antifouling and antibacterial solutions and assessing their antifouling and antibacterial performance and practical feasibility. This review introduces state-of-the-art antifouling and antibacterial materials and solutions for several applications. In particular, this paper focuses on antibacterial and antifouling agents for concrete and cultural heritage conservation, antifouling sol–gel-based coatings for filtration membrane technology, and marine protection and textile materials for biomedicine. In addition, this review discusses the innovative synthesis technologies of antibacterial and antifouling solutions and the consequent socio-economic implications. The synthesis and the related physico-chemical characteristics of each solution are discussed. In addition, several characterization techniques and different parameters that influence the surface finishing coatings deposition were also described.

## 1. Introduction

Fouling is an undesirable phenomenon where macromolecules, microorganisms, or suspended particles adhere to surface materials. This process is a widespread impediment, causing problems in the construction and cultural heritage sector, marine, and industrial applications [1]. Biofouling causes increased energy demands, pipe blockage, decreased efficiency, and water contamination. In marine environments, ship covering biofouling increments drag, corrosion, fuel consumption, and engine stress. There is a general need to find approaches to reject or reduce fouling. The research for efficient antifouling technologies to combat fouling has been ongoing for centuries, and it has been experiencing extensive renovations [2,3,4]. Early formation antifouling systems were produced to be antimicrobial, which required biocidal materials that could eliminate fouling organisms and consequently prevented their settlement. The developed antimicrobial solutions varied from simple lead and copper coverings on wooden boats to antimicrobial coatings containing arsenic, copper, and mercury on ship hulls. Copper was a practical and extensively employed biocide but only showed to be effective for a period of up to two years. When combining biocidal tributyltin (TBT) into coatings, this short lifespan could be prolonged to more than five years. The widespread utilization of metal-based antifouling coatings resulted in high-level contamination and a global prohibition on their usage. Increased knowledge of the negative environmental impact of using toxic biocides stimulated the development of non-toxic, eco-friendly alternatives, including fouling-release coatings that incorporated polymers, oils, and “natural” coatings that included antifouling solutions extracted from organisms [5]. However, natural coatings were hard to commercialize due to the restricted quantity, high cost, short-term efficiency, and specificity of natural antifouling solutions. Furthermore, these coatings still struggled to meet the environmental legislation demands [6]. Instead, the focus shifted to polymer-based coatings, as they succeed in several disadvantages of traditional coatings. Polymer-based coatings are economical, biocompatible, non-toxic, simple to prepare, wide-range efficiency, and highly versatile [7]. Their functionalities and designs can be easily modified, which tunes interfacial and antifouling features. Precisely, polymer sol–gel-based coatings are cited for their ability to alter the characteristics of a surface by producing a nanometric layer. These coating solutions function as a physical barrier between the surface and surrounding foulants in two ways:

(1) If a foulant overtakes the surface, the coating layer will reduce the number of possible formations, entropically unfavorable, consequently inducing steric repulsion and inhibiting settlement;
(2) If a tightly bound hydration layer surrounded the coating layer, water would have to be replaced for an adhering fouling particle. The dehydration process is thermodynamically unfavorable, leading to the repulsion of advancing foulants [1].

These are the principal reasons why research into antifouling and antibacterial solutions has flowered over the last years and demonstrates why this review focuses on these particular kinds of solutions. Furthermore, achieving an effective fouling control method remains a significant challenge in several applications, induced by the reduced mechanical stability and/or short-term antifouling stability of existing antifouling coatings. The latter is partially associated with the great variety of foulants living inside the system of interest. The types of fouling can be classified as follows:

- Organic fouling: settlement of the organic matter, such as proteins, polysaccharides, lipids, etc.;
- Inorganic fouling: inorganic substances, such as salts and metal oxides, result from crystallization or corrosion processes;
- Particulate fouling: growth of colloidal particles;
- Biofouling: settlement of the biological matter, which grows into biofilm microorganisms and leads to macroscopic biofouling [8].

**Figure 1:**
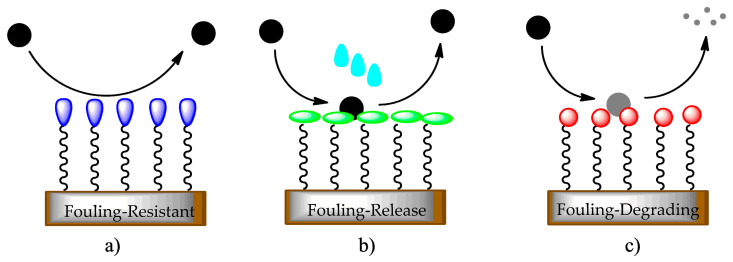
Schematic representation of the three major antifouling approaches: (**a**) limiting foulants from attaching to the covering (fouling-resistant), (**b**) minimizing foulant interaction with the surface (fouling-release), and (**c**) deteriorating/killing biofoulants (fouling-degrading).

Fouling includes more than one foulant or fouling mechanism and is then related to composite fouling. Fouling depends on surface features, such as surface energy and wettability. Therefore, remodeling the surface structure provides a straightforward approach to fouling limitations. A successful process to reach this purpose is by treating the substrate with an antifouling and antibacterial silica-based coating solution. The presence of many varieties of fouling requires different antifouling coating procedures, such as fouling-resistant, fouling-release, and fouling-degrading sol–gel coating solutions (Figure 1).

- Fouling-resistant: inhibits adhesion of macromolecules, microorganisms, or bacteria, usually associated with forming an intensely hydrated surface, as this provides a physical barrier to foulants [9];
- Fouling-release: provides limited foulant–surface adhesion but promotes simple removal of foulants by applying a little mechanical force, such as a water jet or an external trigger [9];
- Fouling-degrading: deteriorates adsorbed foulants through oxidizing agents and/or other microorganisms by bactericidal functionalities [10].

**Figure 2:**
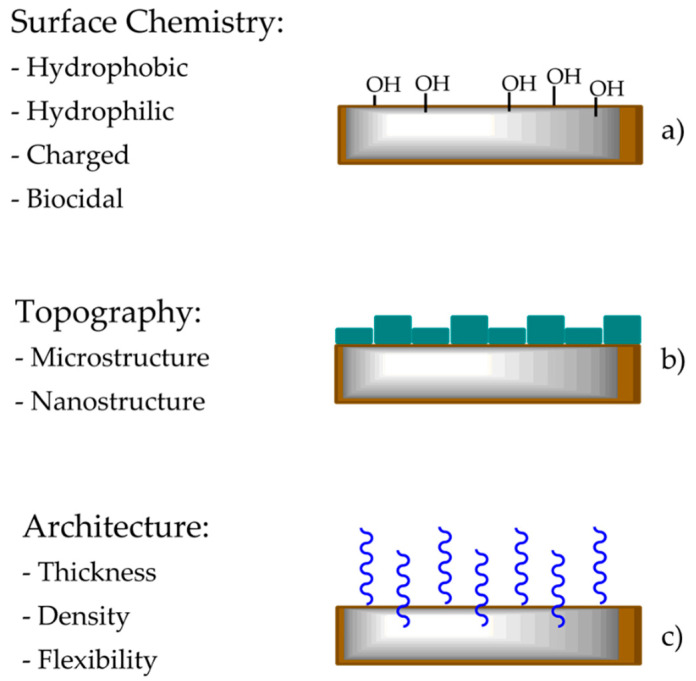
The strategies to provide a surface with antifouling features are (**a**) alteration of surface chemistry, (**b**) surface topography, and (**c**) the coating design.

These antifouling strategies include modifying the surface chemistry, surface topography, and architecture (Figure 2).

Surface chemistry determines the way foulants interact with the surface, which should be hydrophilic, hydrogen bond-forming, and electrically neutral [11]. The antifouling ability of hydrophilic and zwitterionic surfaces is related to the high hydration and surface energy because the tightly bound water layer constitutes a physical barrier, inhibiting adsorption [12]. On the other hand, a hydrophobic surface with lower surface energy provides a higher self-cleaning potential. In addition, the surface charge is also essential to prevent nonspecific adhesion. 

Furthermore, microorganisms can be removed by incorporating charged antimicrobial/biocidal moieties inside the coating. Finally, surface topography can prevent the settlement of microorganisms by imposing size limitations. Microorganisms usually settle in large areas to achieve maximum protection and surface area contact. Consequently, producing a micro or nanostructure on top of the surface can reduce the chances of attachment, limiting foulant adhesion and promoting the eventual removal of foulants. Therefore, this antifouling approach facilitates both fouling resistant and fouling-release coating solutions [2]. The architecture is a relevant strategy that involves the structuring of the coating interior. Foulants adhere in three ways:

v. Primary adsorption: penetrate through and adsorb onto the substrate;
vi. Secondary adsorption: adsorb on top of the substrate;
vii. Tertiary adsorption; adsorb inside the substrate [13].

Tuning the cover design may improve control over the surface development and coverage, implement access to specific functional groups, facilitate the structured surface formation, and restrict the interaction among foulants and the underlying covering [7]. The polymer brush’s grafting concentration, thickness, and adaptability are fundamental parameters that should always be considered while planning such silica-based coatings [14]. Recently, significant effort has been dedicated to producing antifouling Si-containing coatings.

All different materials produced by sol–gel methods present similar advantages:

(a) The thin coating ensures excellent adhesion between the substrate and the top layer;
(b) Protection against corrosion;
(c) Simple, economic, and efficient production;
(d) Highly controlled composition.

The production of these materials employed for several applications also presents disadvantages:

(a) The contraction of the material that occurs during curing and processing;
(b) Presence of residues of unreacted chemicals;
(c) Use of organic solvents, which can be unhealthy.

Future research is focused on optimizing the production process to obtain eco-friendly materials with the desired composition and volume in a shorter time. The focus of this review is to highlight the emerging role of biodegradable polymeric micro and nanostructures that show intrinsic antifouling and antimicrobial properties, such as physical–mechanical, chemical, and electrostatic. Collecting and discussing the updated outcomes in this field would help develop better performing biomaterial-based antimicrobial strategies, which are helpful for different applications. For example, an antibacterial and antifouling solution to enhance technical achievability should satisfy diverse specifications: long-term durability, strength, eco-friendliness, and large-scale applicability, which can be exploited by the use also of different innovative approaches such as PEG-based antifouling surfaces [15], systems that incorporate polymer brushes based on amphiphilic copolymers [16], zwitterionic polymers [17,18] and polyionic liquids [19], or exploiting the advanced properties of micro/nano structural surfaces [20].

## 2. Antibacterial Agents for Concrete

Concrete is the most used material for the construction of millions of structures around the world. Although it plays a crucial role in building development, it is considered an environmental pollutant due to the CO_2_ emissions resulting from its production [21,22]. Furthermore, cementitious structures are often exposed to high humidity environments and the attack of atmospheric agents, such as acid rain, which make them vulnerable to microbial attachment with consequent colonization and deterioration over time. In the light of these considerations, the researchers turned their attention to designing green and sustainable alternative materials that exhibit similar characteristics to the traditional concrete by using nanotechnologies [21]. It is well known that stains on concrete walls and building facades are due to biodegradation phenomena and cement structures for irrigation and sewerage, which usually arise from the growth of cyanobacteria, fungi, and algae [23,24]. The microbial growth and microorganism present on the concrete surface are closely related to pH values, climatic exposure, and nutrient availability [25]. In addition, as previously mentioned, acid rains and air pollution can promote microbial development due to the formation of nitrogen or sulfur-containing compounds [26,27]. Some different mechanisms in which microorganisms can contribute to concrete deterioration are reported [28]. Physical deterioration caused by the bacteria proliferation, which leads to the mechanical breakage of concrete structures, aesthetic worsening due to biofilm formation on building surfaces, and chemical corrosion, deriving from the elimination of metabolites, were considered the main routes of degradation [26]. All these factors that negatively affect aesthetic characteristics, mechanical properties, and the stability of concretes also involve additional costs for repairing and renovating constructions. For these reasons, researchers tried to develop alternative and innovative cementitious materials that could show antimicrobial, antibacterial, and antifouling properties by using additives to the cement paste having antimicrobial properties against one or more microorganisms without affecting the mechanical properties of the concrete material.

All antibacterial agents for concrete protection mentioned in this section are summarized in Table 1.

### 2.1. Polymers and Inorganic Biocidal Additives as Antibacterial Agents

In the past years, scientists tried to reduce bacterial degradation by treating the surfaces of concrete structures with biocidal agents or antimicrobial polymers added directly to the cement mix. In work presented by De Muynck et al. in 2009, metal zeolites and antibacterial polymeric fibers were used on the concrete surface of sewers to prevent biogenic sulfuric acid corrosion [29]. Despite the presence of antibacterial compounds leading to a significant bacterial activity decrease, commercial surface treatments with epoxy and polyuria coatings showed better results. More recent studies performed by Kong et al. in 2019 confirmed the best protective effect of epoxy resins for cement exposed to the corrosion action of wastewater [30]. In their paper, the authors reported the investigations on an epoxy coal tar pitch coating, a cement-based capillary crystalline waterproofing coating, and a cement-based bacterial coating. The first one presented an excellent effect of shielding from wastewaters corrosion. It shows a low porosity structure and the same compressive strength as the untreated sample concrete immersed in water and cement-based biocides coatings, whose copper phthalocyanine, cuprous oxide, and potassium nitrate were functional components. Many other biocide agents suitable for concrete were described in the literature, such as quaternary ammonium compounds, halogenated complex, metal oxide, silver, and tungsten powder [31,32,33].

### 2.2. The Use of Nanotechnologies to Prevent Microbial Growth

In recent years the use of nanotechnologies to control the effect of microbial proliferation on concrete was investigated [50,51]. In particular, CuO, Cu_2_O, ZnO, TiO_2_, Al_2_O_3_, and Fe_3_O_4_ nanoparticles (NPs) incorporated in the cement paste, as reported by Sikora et al., showed biocide activity, although the colonies were able to re-proliferate [34]. In current technologies, silver nanoparticles are also added to the commercial silica-based coating to provide antimicrobial properties to the wall coverings [35,52]. The addition of these inorganic agents to paints, such as ZnO and MgO NPs, as described by Singh et al., promotes protecting the aesthetic properties of building surfaces, avoiding bacterial development [21]. Dyshlyuk et al. showed that zinc oxide nanoparticles with a size of 2–7 nm and a concentration between 0.1 and 0.25% in aqueous suspension decreased the proliferation of microorganisms that commonly attack building materials by 2–3 orders of magnitude. At the same time, TiO_2_ and SiO_2_ exhibited lower bactericidal activity [36]. Another strategy to improve the antimicrobial action, thermal resistance, and durability is developing and incorporating SiO_2_–Ag nanohybrid compounds into acrylic coatings, as Le et al. [37] defined. The use of SiO_2_ particles within the paints has the task of improving the adhesion of coatings to concrete walls of the buildings through chemical interactions with the components of the cement paste. A correct understanding of these interactions, which play a significant role in coatings’ biocidal activity, can help develop materials with improved antifouling properties. Recently, Dominguez et al. synthesized a coating of silver nanoparticles deposited on N-SiO_2_ nanocarriers by using *N*-[3-(trimethoxysilyl) propyl] ethylenediamine and encapsulated in an organically modified silica matrix (ORMOSIL) by using the sol–gel method [38]. The -NHx groups, positively charged, linked to SiO_2_ nanoparticles, lead to the interaction with the cell walls having a negative charge [53]. This coating showed better antifouling properties due to its ability to form a rough surface at the nanometer level, giving it superhydrophobic proprieties. Another study, illustrated by Gao et al., described the importance of hydrophobicity of coatings as a property that affects bacterial activity and as great potential for energy saving in buildings [39]. They synthesized coating based on BiOCl_x_Br_1−x_ micro flowers featured by a high NIR reflectance. Once applied on the surface of building materials, such as concrete, they can decrease microbial growth at the inner temperature [39]. Zhu et al. also described the possibility of synthesizing hybrid silica coatings having, at the same time, heat reflective, antifouling, and weatherable properties. They prepared a superhydrophobic mortar for buildings by mixing black pigments, cement, sand, and TiO_2_ nanoparticles and applied a fluorine silicon sol on the concrete surface [40]. The antibacterial activity of TiO_2_ coatings was also investigated by Verdier et al. They evaluated the resistance of semi-transparent coverings to developing bacterial biofilms under amplified growing conditions on cementitious substrates [41]. Although titanium oxide showed high potential in the construction field, it presented a significant limitation due to its excitation when exposed to ultraviolet radiation. The most valid solution is to modify the TiO_2_ photocatalyst structure with non-metallic elements or dope it with transition metal ions [54]. Janus et al. studied the microbial inactivation on concrete plates treated TiO_2_ modified with carbon and nitrogen, describing an increase in the bacterial removal rate and enhancing the antimicrobial activity [42]. Mortars improved with modified titanium dioxide could be broadly used in buildings, which request high decontamination degrees, such as hospitals, schools, or water storage constructions. Dehkordi et al. suggested using TiO_2_ and ZnO nanoparticles in addition to polyethylene glycol (PEG) as a new antibacterial coating applied on the surface of building materials [43]. PEG, used as a stabilizer to prevent the growth of common bacteria, i.e., *E. coli*, enhanced the stability of the white Portland cement samples under study. It is well known that also iron oxide shows antibacterial activity [34]. Baalamurugan et al. demonstrated that Fe_2_O_3_ contained in steel slag of an industrial induction furnace owns antibacterial activity and can be used to produce construction materials to enhance the resistance against microbial deterioration [44].

### 2.3. Hybrid Geopolymer-Based Materials with Antimicrobial Properties

Fly ashes, a waste product of the thermal power plants featured by a large amount of SiO_2_ and Al_2_O_3_, can also be exploited to produce innovative materials with antibacterial properties. In this regard, Rodwihok et al. reported a study where fly ashes were recycled by an alkali activation process supported with Zn, which enhanced the microbial growth inhibitory properties [45]. These waste materials can also be used as primary resources to prepare and synthesize innovative hybrid geopolymer-based materials. These matrix components with strong antibacterial properties can be added, thus developing new protective concretes for buildings. Recently, in the literature, the antiseptic efficiency of metakaolin-based geopolymer cement loaded with organic and inorganic compounds, such as 5-chloro-2-(2, 4-Dichlorophenoxy) phenol and glass waste towards Gram-positive and negative bacteria, was described [46,47]. Another critical aspect of being assessed concerns concretes and cements relating to the masonry structures of buildings and those about civil infrastructures and urban wastewater drainage systems. In this regard, Roghanian et al. studied three types of coatings to prevent bio-corrosion in wastewater pipes due to the formation of sulfuric acid by microorganisms [48]. They investigated the effect on microbial growth by applying different coatings between the concrete and the pipes surface obtained from the adding of zinc particles or zinc doped clay particles, demonstrating a higher resistance to the degradations concerning the ordinary cement-based and geopolymer-based coverings. Justo-Reinoso et al. suggested replacing fine aggregates traditionally used in cement mixtures with granular activated carbon and fundamental oxygen furnace steel slag particles, copper, and cobalt as inhibitory metals towards the acidogenic microbial development in concrete sewer pipes [49]. This study also described improved mechanical properties, such as compressive and flexural strength for the cement-treated, concerning the conventional ones.

## 3. Antifouling and Antibacterial Agents for Cultural Heritage

A significant challenge for cultural heritage conservation science concerns the development of innovative, low environmental impact, and eco-sustainable customized protocols [55]. Green conservation of cultural heritage concerns all eco-friendly methodologies used in preserving and restoring cultural heritage as an alternative to the use of products that are often toxic to humans and the environment. In order to face the problem of biodeterioration of cultural heritage, resources are necessary to understand the causes of the growth of macro and/or microorganisms. Consequently, it is essential to promote proper procedures to slow down or eliminate undesired biological growth [56]. Developing a sustainable conservation procedure is necessary to determine the bioreceptivity, which is the ability of a given material to be colonized by living organisms. This procedure depends on parameters such as the material composition, surface treatments, conservation status, and environmental conditions [57]. Different methods were used to block the growth of unwanted colonies based on various factors, including the nature and state of conservation of the artifact, colonization types, and degree of extension.

The chemicals usually used in the restoration of cultural heritage are versatile. Most of them were pesticides or herbicides used in agriculture [58]. They must also be non-aggressive and economical products. However, the European Community has introduced significant restrictions on using chemical biocides potentially harmful to humans and the environment. Chemical products suitable for the coating of artifacts are generally a combination of consolidating agents, water repellents, and surfactants [59]. There are essentially two approaches for applying the antibiofilm or antifouling coating. The first method employs chemically active antimicrobial coatings, while the second causes the inhibition of biofouling colonization without carrying out chemical reactions [60]. Using natural or biological substances with biocidal activity is extremely interesting for conserving cultural heritage [61]. These natural substances have several origins, such as zoosteric acid and capsaicin, which are examples of microbial by-products, extracellular enzymes, hydrolases [62], usnic acid and parietin, and plant extracts such as essential oils [63], each substance can be used alone or incorporated into a sol–gel matrix to improve the overall performance [64]. Currently, there is a lack of information on the specific efficacy of the biodeteriogen based on the concentration used, the duration of treatment, or any interference with the artifact’s material [65]. The protocol implemented to combat biodeteriogens consists of two actions. First, it is necessary to remove the existing biomass using conventional biocides that act on a broad spectrum of organisms. Thus, they can be washed off without damaging the product and without interfering with subsequent surface treatments. After the biofouling removal phase, it is necessary to prevent recolonization [66]. Several biocides were recently developed to be used alone or in a mixture to protect stone cultural heritage. Despite recent advances, the most widely used formulations are only conventional ones based on quaternary ammonium salts. These solutions increase efficiency, stability, and range. 

The products most used for the restoration and conservation of cultural heritage are listed in Table 2. Despite their known toxicity, these compounds are used at low concentrations to significantly reduce the risks for humans and the environment [67]. The efficacy of these biocides is related to their spectrum of action.

Based on conventional conservation treatments, the effectiveness and duration of three types of stone material treatment were compared [69]:

1. In the first case, the simple mechanical removal of the biodeteriorants was carried out, and it was observed how the recolonization of the stone occurs rapidly;
2. In the second case, Rocima was used to remove the already existing biofouling followed by microwave treatment. In this case, the biocidal activity lasted for five years;
3. In the third case, the stone material was treated with a mixture of Biotin R, in 5% ethyl alcohol, used alone or mixed with Titania nanoparticles and Silver–Titania core-shell nanoparticles (TiO_2_ and Ag-TiO_2_). This treatment resulted in a prolonged biocidal action up to 8 months after application.

Producing new antifouling materials is essential to select either renewable or eco-sustainable raw materials in compliance with Life Cycle Assessment (LCA) standards [70].

Nanotechnologies provide antibacterial and antifouling innovative and eco-sustainable methodologies for Cultural Heritage conservation [71]. In particular, recent approaches based on nanoparticles contributed some promising alternatives to protect damaged Cultural Heritage. Nanoparticles such as Au, Ag, SiO_2_, ZnO, TiO_2_, Mg(OH)_2_, Ca(OH)_2_, ZrO_2_, and TiO_2_ were combined with appropriate precursors and applied in stone material restoration [71]. In addition, NPs provide consolidation and protection of the substrate. For example, the combination of polymer matrices with low surface energy and nanoparticles produce biomimetic nanostructured surfaces with controlled wettability and roughness [72]. TiO_2_-based materials are currently employed as self-cleaning and antibacterial solutions, but near-ultraviolet irradiation is required for photocatalytic activation. The use of nanotechnologies in Cultural Heritage has raised severe concerns regarding human health and environmental risks, efficiencies against microorganisms, and long-term effects on the material [73].

## 4. Antifouling Coatings for Filtration Membrane Technology

**Figure 3:**
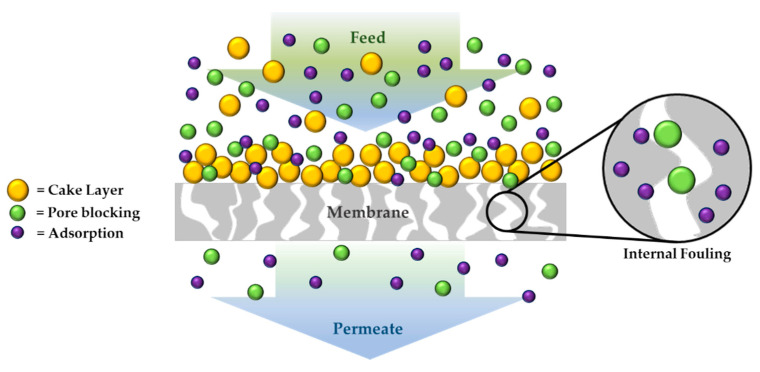
Different mechanisms of membrane fouling.

With the increase in the world population and the demand for drinking water, developing new wastewater treatment technologies or improving existing ones is necessary. Filtration membranes technology is a pressure-driven process widely used to reject different pollutants and/or desalination water. In particular, it is possible to distinguish four major categories of membranes with a different contaminant removal capacity depending on the pore size of the membranes; for example, microfiltration membranes (0.1–5 µm pores size range), ultrafiltration membranes (0.01–0.1 µm pores size range), nanofiltration membranes (0.001–0.01 µm pores size range) and reverse osmosis membranes (0.0001–0.001 µm pores size range) [74]. One of the biggest problems of membrane technology is represented by fouling (Figure 3) caused by agents present in wastewater such as inorganic compounds, bacteria, proteins, and other organic molecules. Reversible and irreversible fouling in water treatment processes decreases the membranes’ efficiency and lifespan, causing a high energy consumption since it is necessary to use higher working pressures, and therefore economic damage. UF and RO membranes are mainly affected by external fouling due to their dense structure, while internal fouling affects mainly MF and UF membranes characterized by larger pores [75].

An approach to avoid fouling concerns membrane surface modifications; in this regard, some of these innovative approaches are reported in Table 3 and are explored below.

Surface hydrophilization with poly(ethylene glycol) (PEG), one of the most representative hydrophilic polymers, effectively prevents fouling due to forming a water layer that avoids the interactions between the foulant and the membrane surface. Antifouling coatings also affect the adhesion of the foulant on the membrane caused by electrostatic interactions. For this purpose, zwitterionic coatings represent a solution to obtain minimal adhesion on the membrane surface and prevent fouling [90].

Different techniques were used for the preparation of zwitterionic polymeric coatings, such as self-assembled monolayers (SAMs) [79], layer-by-layer deposition methods (LBL) [86], solution polymerization, solvent evaporation, and atom transfer radical polymerization (ATRP) [78]. A simple approach to incorporate zwitterionic copolymers in polymer membranes, consisting of electrostatic adsorption, performed a dip-coating method. Zwitterionic copolymers were synthetized by a radical polymerization from [2-(methacryloyloxy) ethyl] dimethyl-(3-sulfopropyl) ammonium hydroxide (SBMA) and [2-(methacryloyloxy) ethyl] trimethylammonium chloride (MTAC), for the modification of a negatively charged poly(vinyl chloride-co-acrylonitrile-co-sodium 4-styrenesulfonate) (PVC-PAN-PSS) membrane by a dip-coating approach. A stable film was obtained with anti-organic fouling and anti-biofouling properties due to the improved hydrophilicity and reduced negative charge of the membrane surface [76]. For this purpose, innovative techniques were also developed that prevented the damage of delicate substrates, such as initiated chemical vapor deposition (iCVD). This last is an all-dry free-radical polymerization technique working at low temperatures and operating pressures. Furthermore, iCVD was synthesized from a thin film made of poly [2-(dimethylamino)ethyl methacrylate-co-ethylene glycol dimethacrylate] (PDE) and reacted with 1,3-propane sultone to obtain the zwitterionic structure. The thin films were deposited on commercial RO membranes that show superior antifouling performances and low cell adhesion of *Escherichia coli* than the bare RO membrane and not impaired salt rejection performances [77].

Another approach for synthesizing hydrophilic and zwitterionic thin films consists of plasma polymerization and surface-initiated atom transfer radical polymerization (si-ATRP). Antifouling coating for desalination RO membranes was prepared by a combination of plasma activation, plasma bromination, and si-ATRP of hydroxyethyl methacrylate (HEMA), 2-methacryloyloxyethyl phosphorylcholine (MPC), and [2-(methacryloyloxy)ethyl]-dimethyl-(3-sulfopropyl)ammonium hydroxide (SBMA) [91]. The obtained coated membranes show an improved permeation flux and an increased resistance towards the adhesion of *Pseudomonas fluorescens* with a biofilm reduction of 85.4% for the MPC-coated membrane [78]. Ultrathin monolayer and bilayer coatings to prevent the oil fouling in UF membranes were prepared by the self-assembly of star-shaped block copolymers (SPs). A hydrophobic ultrafiltration (polysulfone, PSF) membrane was coated with SPs, consisting of a hydrophobic polystyrene (PS) core and different types of hydrophilic arms, including polyethylene glycol methacrylate (PEGMA), polydimethylaminoethyl methacrylate (PDMAEMA), and polymethacrylic acid (PMAA). These coated membranes, by a self-assembly process, were used for filtration of synthetic oil-water emulsions. In particular, the bilayer-coated membrane showed an 80% and 90% retention capacity for nano and micron size oil emulsion, respectively [79]. An alternative to zwitterionic polymer coatings is represented by polydopamine (PDA) based coatings that are becoming increasingly popular for the surface modification of different substrates due to their versatility, adhesion capacity, and reactivity due to the quinone group on PDA that can be easily functionalized with different molecules. For example, a PES ultrafiltration membrane PDA coated by a dip-coating process for the subsequently grafting of fluorinated polyamine to the PDA layer through Michael’s addition reaction. As a result, the PDA reduced the pore size of the PES membrane and provided the active sites for the grafting of fluorinated polyamine. The obtained membranes also showed excellent antifouling properties and fouling-release property and a rejection of Congo red above 99% (flux about 46 L/(m^2^ h) under 0.1 Mpa) [80].

Hybrid coatings represent an innovative approach for the surface implementation of functional properties of different substrates. In this concern, polysulfone (PSF) ultrafiltration membranes were coated with a polydopamine–Zn complex, formed by in situ polymerizations of dopamine in the presence of zinc species, to improve their stability in both highly acidic and alkaline solutions, the water flux permeability, and the antifouling properties [81].

The antibacterial properties of some nanomaterials, such as Ag nanoparticles (Ag NPs), are exploited to synthesize these hybrid coatings. For example, Tannic acid inspired antifouling, and the antibacterial membrane was prepared through the co-deposition of zwitterionic polymers and AgNPs. A poly(ether sulfone) (PES) membrane was coated via a dip-coating process with tannic acid (TA), Ag^+^ ions in situ were reduced by the TA layer, and a zwitterionic polymer was obtained via the quaternarization between polyethyleneimine (PEI) and 1,3-propane sulfonate. These membranes prepared by an economical, environmentally friendly, and universal method showed a long-term and robust bactericidal activity and resistance to bacterial adhesion [82]. Another example is represented by a mussel-inspired method that exploits the adhering capacity of catechol moieties, their reactivity with the –NH_2_ groups, and the capacity to reduce and Ag^+^ ions into Ag NPs chitosan-polyurethane coatings loaded with AgNPs for PES membranes. For those purposes, the o-carboxymethyl chitosan (CMC) was directly reacted with catechol and loaded with AgNPs via in situ reductions. Subsequently, a PEG-based polyurethane (PU) was used for the preparation of CMC–Ag–PU composite coating to confer PES UF membrane with antifouling and dual-antibacterial properties (*E. coli* and *S. aureus*) [83]. Chitosan-coated PES membranes can also be coated with thin films made of porous metal–organic frameworks (MOFs) to improve their permeability, removal efficiency, and antifouling activity. Furthermore, a novel membrane with antibacterial activity was prepared via in situ rapid formation and deposition of copper (II)-benzene-1,3,5-tricarboxylate (Cu-BTC) clusters on chitosan-coated PES membrane surface, this last obtained by a dip-coating process for the sustainable removal of manganese. The Cu-BTC/CS membrane showed, in fact, an inactivation capacity of 83% of *Escherichia coli* bacteria [84]. Coatings based on photocatalysts such as ZnIn_2_S_4_ to form a photocatalytic layer on the membrane surface can represent a different solution for membrane fouling. Moreover, the photocatalyst ZnIn_2_S_4_ was deposited on the surface of a polyvinylidene fluoride (PVDF) membrane through dead-end filtration, obtaining a dynamic photocatalytic membrane with a loading of 2.6 mg/cm^2^ showing high stability, outstanding antifouling property, and removal efficiency of fluvastatin and TOC of 97.19% and 53.29%, respectively [85].

Chitosan is not the only bio-polymer used to improve the surface properties of different supports. There are many examples in the literature of bio-based functional and antifouling coatings for membranes. Another renewable, abundant and biodegradable biopolymer is nanocellulose, which consists of aligned molecular chains of high crystallinity. An LBL deposition technique makes it possible to coat membranes with nanocellulose to change the surface characteristics and mitigate the fouling. Therefore, a commercially PES microfiltration membrane was coated with nanoporous and nano-textured layers composed of cellulose nanocrystals (CNC) or TEMPO-oxidized cellulose nanofibrils (T-CNF), resulting from the electrostatic interactions between the PES substrate, polyallylamine hydrochloride (PAHCl) anchoring layer, and the nanocellulose functional layer. The obtained coated membranes exhibited up to 49% less relative adhesion of bovine serum albumin (BSA) than the uncoated membrane, increasing wettability (up to 52%). In particular, the T-CNF coated membrane demonstrated significantly enhanced antifouling and antibacterial properties for *E. coli*, compared to CNC, attributed to the pH reduction effect induced by the carboxyl groups [86]. Agricultural biomasses such as lignin, the second most abundant biopolymer in nature, are also used to modulate the hydrophilicity and antifouling properties, permeability and antioxidant activity, thermal stability, and adsorption properties of membranes. In this context, by an LBL method, a hydrophilic sulfonated kraft lignin, abundant in anionic functional groups, was used to coat the surface of a PES UF membrane, using poly (diallyldimethylammonium chloride) (pDAC) as polycation. For the treatment of oily wastewaters, the n-hexadecane-in-water emulsion was used as synthetic oily wastewater. The best performances, such as the highest surface hydrophilicity (22.6° ± 0.5° water contact angle), and the highest fouling resistance (23% flux decline and 93.8% FRR), were obtained by the membrane prepared with three bilayers of pDAC/lignin from a 2 wt% concentration of the respective solutions [87].

Silica-based sol–gel coating processes can also be exploited for the preparation of membranes with antifouling properties. In this regard, a PVDF membrane was coated with a novel synthetized zwitterionic organosilica polymer (poly-BPGH) by the use of a sol–gel process and a filtration cell. A smooth and uniform organosilica xerogel coating was obtained on the membrane surface that showed an anti-bioadhesion ability (tested with test with *E. coli*) and a flux recovery rate of 67.76% and 90.66%, for BSA and sodium alginate, respectively, after three cycles of fouling [88]. Another example is represented by a bio-inspired superhydrophilic nanocomposite membrane, consisting of a PVDF membrane coated with a facile and eco-friendly process by polydopamine anchored SiO_2_. In particular, the PVDF membrane obtained by a phase-inversion process was functionalized with polydopamine and then loaded with SiO_2_ nanoparticles obtained by an eco-friendly sol–gel method from TEOS. The obtained nanocomposite membrane, as a result of the surface micro-nano structure and a pore induced capillarity phenomenon, showed superhydrophilicity/underwater superoleophobicity properties and antifouling oil and high-efficient oil-water emulsion separation capacities with high efficiency (>98%) after 10 cycles of oil-water emulsion separation and regeneration [89]. Therefore, several efficient and innovative approaches and systems were reported for the production of antifouling, antibacterial, and anti-oil fouling silica-based coatings mainly based on the use of zwitterionic polymers, mussel-inspired procedures, nanomaterials, and biopolymers, intended for filtration membrane technology to lower the costs and energy consumption of wastewater treatment for more sustainable and efficient processes.

## 5. Antifouling and Foul-Release Coatings for Marine Applications

Accumulation of living organisms on artificial surfaces by adhesion and subsequent proliferation is often the initial step leading to biofouling formation. Preventing bacterial growth associated remains a significant challenge in marine industry applications [92,93,94,95]. This aspect constitutes a problem that must be countered and controlled when the need arises to have efficient surfaces from the hydrodynamic point of view (e.g., boat hulls, pipes). There are more than 4000 species of “foulers”, each with its characteristics. There are two large groups into which the organisms that cause this phenomenon can be divided, and the fouling process is usually divided into four phases. The two main categories into which it is possible to subdivide are: “microfouling”: characterized by very small foulers (micrometer size), they form the famous “slime” mainly formed by sea mold, diatoms, and unicellular organisms. They affect the resistance to the motion of the ship up to a maximum of 10%. “Macrofouling”: bulkier foulers (reaching thicknesses of several centimeters) than the micro category, it includes algae of considerable size and animal fouling. They can affect the resistance to the motion of the ship by up to 40%.

Biofouling is an ecological succession in which microfouling, consisting of bacteria, unicellular algae, and cyanobacteria, establishes itself on the surfaces and prepares them for the attack macrofouling. This settlement consists of larger marine organisms of both vegetable (macroalgae) and animal origin (serpulids, barnacles, bivalves, sponges, and more) [96]. The phenomenon begins to occur when the ship is immersed in marine waters. In the first phase, organic material and molecules such as polysaccharides, proteins, and protein fragments accumulate on the hull. A few hours later, the second begins the phase, which sets the stage for subsequent ones. In fact, in this phase, a thin microbiotic film of bacteria and unicellular organisms such as diatoms is formed, creating a solid base for establishing macrofouling. This layer significantly affects the ship’s performance, increasing the resistance to motion between 2% and 10%. The presence of adhesive exudates and the roughness caused by the irregular microbiotic colonies thus favors the settlement of many other particles and organisms, thus starting the third phase. Above all, algal spores, fungi, and marine protozoa are established. Finally, the fourth and final phase of the fouling formation process is enormously intensified, and mainly macro-algae grow on the hull, such as green algae (*Enteromorpha*) and brown algae (*Ectocarpus*). These have extraordinary reproductive potential and strong resistance to widespread environmental fluctuations, especially concerning salinity and dryness, making their detachment complicated. Another protagonist of this phase is animal fouling consisting mainly of barnacles, mollusks, bryozoans, and tubificids [97]. Some adverse reactions of biofouling are [98]:

- High fuel consumption because of the increased resistance due to biofouling, making the hull rougher and the ship heavier. It was proven that microfouling could increase fuel consumption by up to 18% and reduce the sailing speed by at least 20% [99];
- High maintenance costs because drydocking operations need to be performed more frequently [100]. More pollution since cleaning processes generate a large number of toxic substances that are discharged into the ocean;
- Increased ship hull corrosion since the protective coating surface deteriorates because of biological processes. The hull surface is more susceptible to corrosion and discoloration [101].

**Figure 4:**
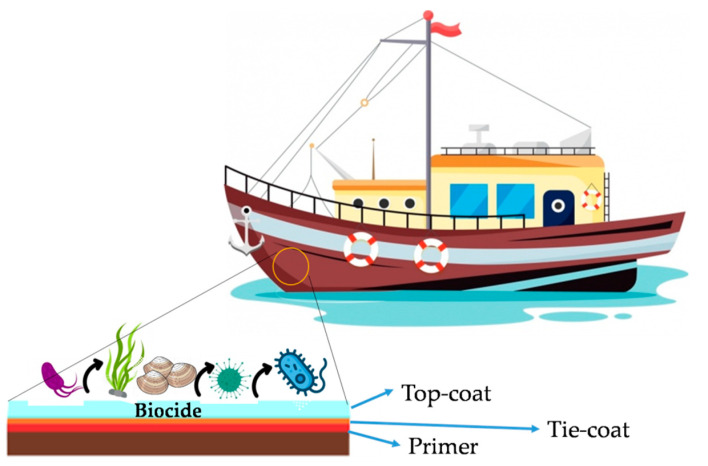
Antifouling topcoat based on molecules with biocidal action.

The development of marine biofouling depends on multiple factors such as water temperature, nutrient level, frequency of currents, salinity and pH of the marine environment, and the hull material’s properties. In addition to environmental factors, surface properties such as surface energy, wettability, mechanical strength, and surface topography are also affected. For example, several studies showed that a surface with energy values between 20 and 30 mJ/m^2^, known as the “Baier minimum”, represents the minimum adhesion condition for microorganisms. Therefore, marine antifouling paints must have high efficacy and guarantee at least five years of constant protection. In addition, paints must also have, in most cases, a broad spectrum of action to be able to counteract the more than 4000 species existing in every environmental and superficial condition in which they can be found. Therefore, each ship will have its antifouling paint most suitable for its purpose and its environmental, speed, and cargo needs. Antifouling paints should contain molecules with biocidal action (Figure 4) released at different times and concentrations depending on the matrices in which they are incorporated to counteract the attack of organisms with strong adhesive capabilities.

Some substances with biocidal action and high efficacy used over the years have shown high toxicity levels in the various sectors of the marine environment. An example is given by organotin compounds (e.g., TBT), whose use as antifouling was prohibited following the indications of the IMO and the international convention (AFS) adopted on 5 October 2002 by the member states of the European Union. Recently, paints based on copper compounds have been banned in Sweden to date the most used. An alternative to paints containing biocides could be the use of polymers with fouling-release action, whose action does not prevent the formation of biofouling but facilitates its detachment due to the weak interactions created between the matrix the structures of membership of organizations.

Biofouling occurs for both physical and biochemical reactions. Physical reactions are governed by factors such as electrostatic interaction and water flow, which lead to the formation of biofilm and the absorption of microorganisms. Biochemical reactions include the adhesion of microorganisms, biofilm formation, and macrofouler adhesion. While physical reactions are generally reversible, biochemical reactions are irreversible. Therefore, it would be easier to prevent biofouling during physical reactions rather than biochemical reactions. Furthermore, successful inhibition of physical reactions would limit subsequent biochemical reactions. Current antifouling research is focused on inhibiting the adhesion of diatoms and bacteria from preventing biofilm formation, although such research has also encountered numerous obstacles [98]. Biofouling is a huge problem for marine industries, and in these years, research was focused on developing an effective antifouling solution. In this review, different kinds of marine antifouling technologies are proposed according to the adhesion mechanism. After the mid-nineteenth century, the most widespread antifouling paint included biocides or toxic substances applied to the hull to kill micro and macro-organisms. Most paints were based on tributyltin compounds (TBT), which guaranteed efficacy on a wide range of species. This painting, however, sterilized some marine species, which did not reproduce and risked extinction. For this reason, TBT technology was banned in November 1999 by the IMO with the ban on the application of paints with organotin compounds from 1 January 2001, and the total ban from 1 January 2008. In the years following the IMO ban, there was a shift to new copper-based release technologies. However, there were environmental problems because the metals present in the paints bioaccumulate in the environment, creating sediments that can be toxic at high levels and permanently hidden for many years. Furthermore, the consequences that biocides generate in the environment are not yet fully understood and are under continuous research. Thus, the age of organotin compounds ended. However, in addition to eliminating the fouling that had already settled on the hull, it was also necessary to prevent its formation by ensuring a certain level of smoothing to minimize the ship’s friction to motion. Therefore, finally, we reach the recently discovered and future coatings called “foul release” and “biocide free”, which offer an excellent compromise between high respect for the environment and efficient ones.

These paints are composed of functional polymer chains released on the submerged surface of the ship’s hull, thus not releasing potentially toxic biocides that are still under study and, therefore, still an unknown factor. In addition, polymer chains inhibit biofouling and biocorrosion more due to their incorporation of anti-adhesion, anti-microbiotic, and anti-corrosion functional chains than the release of biocides. Several coatings and paints based on functional polymers have been developed in recent years, as reported in Table 4, and others are in development, but the three most used types are:

- Biocide-release coatings: based on the dispersion of biocides with different types of polymeric binders released over time in seawater. Currently, these coatings are the most used;
- Fouling resistant coatings: prevent the attachment of “foulers” to the surface;
- Fouling release coatings: reduce the adhesion between marine organisms and the materials of which the submerged surfaces are made;
- Fouling degrading coatings: inhibit/kill “foulers”.

Biocide-based paints are the most widely used among commercial antifouling coatings. They can be divided into four categories depending on the release mechanism:

- Contact leaching coatings (insoluble matrix): the polymer matrix is insoluble in water, while the toxic substances or biocides are incorporated into the paint and released gradually, leaving free pores that are freed by the passage of water that dissolves the toxic particles; however, the matrix remains intact. In this case, the biocides are released at a rate that decreases over time, reducing the effect of the protection. They have a short life (12–24 months), and this has greatly limited their use even if, over time, the duration and the release rate were made more durable;
- Controlled depletive polymer (CDP) coatings (matrix soluble by hydration): in this case, the matrix is composed of biocides and a resin-based soluble matrix that, with the passage of water, are dissolved and released on the surface to protect and contrast the “foulers”. The release process is more controlled than in the previous case and is based on the hydration and dissolution mechanism of the soluble binder. It has a constant and controlled release with a duration of about 36 months;
- Self-polishing copolymer coatings: SPC paints use an acrylic or methacrylate polymer matrix. The release mechanism of the biocides is based on the dissolution of the matrix by hydrolysis in seawater, which gives a considerable smoothness to the surface, thus reducing friction and, therefore, the resistance to the ship’s motion. In addition to making the surface smooth, decomposition by hydrolysis also detaches organisms from the hull, releasing biocides. The release rate of substances can be controlled based on the degree of polymerization and the polymer chains’ hydrophilic properties. Paints with modern SPC technology have five years and occupy 80% of the antifouling paint market;
- CDP and SPC mixed coatings: I combined the properties of the last two technologies mentioned. The dissolution of the matrix is obtained both by hydrolysis and by hydration with better control of the biocide release rate and a duration of 5 years.

Recent research has focused on finding alternatives or changing structures, such as zwitterions. The zwitterionic polymer coatings have been extensively used for anti-biofouling. It can be attributed to the hydration layer around the zwitterionic moiety [102]. According to Saffarimiandoa et al., zwitterionic sulfobetaine silane coatings showed a strong antibacterial effect against the isolated marine biofouling bacteria and significant biofilm adhesion resistance [108]. Bodkhea et al. showed that the amphiphilic and zwitterionic groups on the surface of fouling-release coatings could improve the coatings’ antifouling performance. However, Zwitterion’s monomers can be expensive, and there are definitely difficult to use in organic solvents in addition to being unstable during polymerizations [103,109].

Non-biocidal commercial paints, on the other hand, range from tough matrix paints used mainly for small boats and often challenging to clean up to paints with “foul release” or release technology that has the property of making the surface of the hull with low energy making it so easy to clean and making organisms detach easily without the use of biocides. The “foul release” technology coatings are based on the concept of minimizing the adhesion force between the foulers and the material of which the hull surface is made, allowing the removal of the fouling simply through the motion of the ship, so it is a type of cleaning machines that exploit the hydrodynamics of the ship during navigation. Silicone polymers and fluoropolymers with low surface energy and modulus are the most used materials as binders. Hydrophobic coatings, e.g., silicone or fluorine-based coatings, have low surface energy. Webster and co-workers developed a self-stratifying coating system consisting of a polyurethane resin tethered combined with a siloxane surface resin [110,111,112,113]. The polyurethane provides desired toughness and adhesion, while the siloxane moiety provides the low-surface energy needed for good fouling release properties. The combination of a polyurethane component for strength in combination with siloxanes or fluoroalkyl compounds to lower the surface energy is widely investigated by various research groups [104,105,106].

Marine fouling organisms and natural organic matter commonly found in seawater usually have negative charges, making them susceptible to electrostatic repulsion by a negatively charged film’s surface. Conductive antifouling coatings are an electrochemical antifouling alternative due to an increased electrostatic repulsion between the films and the foulants, reducing the fouling adhesion [107]. This new technology does not produce toxic substances, which is an essential environmental protection antifouling technology. Mostafacei et al. mixed conductive polyaniline (PAni) with epoxy resin and injected nano zinc oxide as an additive to synthesize conductive nanocomposite coatings (PAni–ZnO) [114].

According to the studies, conductive polymers such as PAni and PAni–ZnO nanocomposite can reduce the settlement of algae and barnacles on the substrate. In summary, contemporary environmentally friendly antifouling technologies all exhibit a significant antifouling performance compared to the coating containing only cuprous oxide, even if many limitations also accompany them. In future research, environmentally friendly antifouling technology needs to be optimized by gradually combining existing technologies and materials to replace toxic metals coatings.

## 6. Development of Antibacterial and Antifouling Innovative and Eco-Sustainable Solutions from Marine Areas Protection to Healthcare Applications—Textile Materials

Since ancient times, textile materials have played a key role in advancing human society and culture. While in the past, individuals tended to use dress mainly to define themselves, show their power, or exert control over others, currently, due to technological development, the perception about these materials has changed. The end use of textiles is not limited to traditional applications, namely clothing. Still, it includes technical sectors, such as medical and healthcare, protective clothing, smart-textiles, food industry, automotive, geotextiles, agro textiles, fishing equipment, and sportswear [115,116]. Because of these diverse applications and the closeness of fabrics to human skin, it is requested that they possess additional functionalities. Among them, antibacterial capability has become one of the most vital for high-value textiles. Despite many endless applications, it is noteworthy to mention that these materials provide suitable conditions such as moisture, temperature, and nutrients required to grow and propagate pathogenic microorganisms [117,118]. In turn, this inflicts a series of unwanted effects on the fabric itself, such as unpleasant odor, stain, discoloration, reduction in mechanical strength, and the wearer, namely skin infection, allergic reactions, and other related diseases [116,119]. Since the beginning of humanity, several attempts have tackled this issue. An example of the earliest antimicrobial treatments dates back to the ancient Egyptians, whose common habit was to use herbs and spices to preserve mummy wrapping. More recently, two types of fabrics, namely bacteriostatic and bactericidal, based on a different mode of action, were developed. While bacteriostatic textiles discourage the attachment and growth of microorganisms, restricting only the growth of microbes that would enter in touch with a fabric surface, bactericidal fabrics possess the ability to kill the bacteria attached to the surface and surroundings. Because of the outstanding properties of bactericidal fabrics, they are the most commonly available in the antimicrobial textile market.

**Figure 5:**
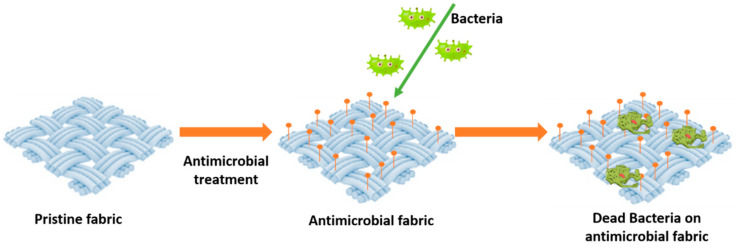
Mode of action of antimicrobial textiles.

Different chemical or physical approaches are available to confer antimicrobial property depending on the fibers’ features, morphology, composition, and surface texture. These approaches can be essentially grouped into two categories: the first consists of incorporating antimicrobial agents into the polymer solution before extrusion; the second allows applying the active ingredient as a layer both on ready-made natural and synthetic fabrics. Finishing techniques can impart beneficial properties to a greater variety of textiles incorporating antibacterial substances in natural fibers [120,121]. The schematic mode of action of fabrics treated with antimicrobial agents is shown in Figure 5.

Although in the previous decades, the main feature required for an antimicrobial agent for textile applications was killing undesirable microorganisms, thus avoiding the spread of diseases. Currently, an antibacterial finishing has to meet four criteria that can be summarized as follows [122]: (1) be effective against a broad spectrum of bacteria, fungus, and molds, but presenting low toxicity and being non-allergic to human users, with good results in compatibility tests (cytotoxicity, irritation, and sensitization) before marketing; (2) be durable to washing, dry cleaning, and heat-press; (3) not negatively affect quality, comfort, or appearance of the textiles; (4) be cost-effective but environment-friendly and not harmful to humans who work on their manufacture [115].

### 6.1. Sustainability Issues of Commercial Antimicrobial Formulations

**Figure 6:**
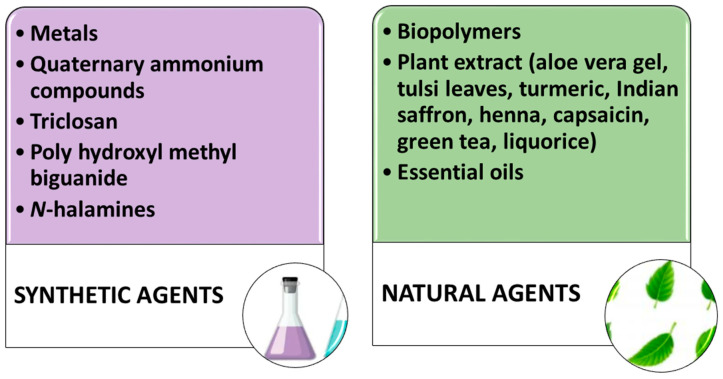
Common antimicrobial textile finishing agents.

Currently, mainly paints based on metals, quaternary ammonium compounds, triclosan, poly hydroxyl methyl biguanide, and *N*-halamines are commercially used for this purpose. These synthetic products are highly effective in killing a wide range of undesirable microorganisms. Some recent research studies showed that most of them can also kill harmless microorganisms and may even be toxic to humans with negative consequences on the environment because of the release of harmful molecules, especially during the laundering process [123]. The active antimicrobial substances and the processing techniques need to be selected by focusing on the aforementioned factors. Furthermore, significant efforts were made to find more eco-friendly and efficient alternatives [118]. For this reason, the use of nanotechnology and the use of nature-derived bioactive materials, such as biopolymers, plants extract, and essential oils, has gained increasing interest among the scientific community. These antimicrobial agents are summarized in Figure 6. Developing an effective green technology for obtaining antimicrobial fabrics is the ultimate challenge to manufacturers and researchers. Currently, nanotechnology is bringing a new revolution in several fields, including the textile industry. The growing interest in nanoscience is mainly due to the outstanding properties of nanoparticles. Their large surface area to volume ratios, high surface energy, and better affinity to the fabrics compared with the bulk material ensure the long-lasting effect of functional activity on textiles. Many research works demonstrated the successful incorporation of various nanoparticles in fabrics, and advantageous properties, such as water repellency [124], self-cleaning [125], flame retardancy [126], bacterial resistance, [127] and UV light protection [128], were conferred to these materials. In the last decades, metal-based nanoparticles have been used in a new class of biocidal formulations. Their synthesis and immobilization in the fabric polymer matrix were achieved by different approaches, such as electrochemical method and thermal decomposition sol–gel, and in situ chemical reduction methods [129].

### 6.2. Sol–Gel Technology: Synthesis of Antimicrobial Formulations

Among the procedures mentioned in the previous paragraph, the sol–gel technique and in situ application method represent the most employed approaches for the antimicrobial treatment of textiles [130]. Tarimala et al. [131] used nanotechnology to modify cotton with dodecanethiol-capped silver nanoparticle-doped silica sol to confer antimicrobial properties to the fabric. The sol was prepared with tetraethyl orthosilicate as the precursor, and dodecanethiol-capped silver nanoparticles were added. The sol was then applied to the fabric by dipping it into the solution and passing through a finishing padder, followed by drying and curing. The results illustrated that the fabric treated with the sol doped with dodecanethiol-capped silver nanoparticle shows an inhibition rate of 40% against the *Escherichia coli* compared to untreated cotton fabric. The sol–gel method was used by Jun et al. [132], who prepared a silver-doped silica thin film. Si(OC_2_H_5_)_4_, AgNO_3_, H_2_O, and C_2_H_5_OCH_2_CH_2_OH are mixed in 1:0.24:3.75:2.2 molar ratios, the pH value of the solution was 3, after adjusting with 0.5 N HNO_3_. The formation of silver-doped glassy silica thin film under different temperature conditions was investigated. The results revealed that the silver ions were fully embedded in the silica matrix, and at 600 °C annealing temperature, their reduction can be achieved. Furthermore, the film showed amazing antibacterial effects against *Escherichia coli* and *Staphylococcus aureus*. Poli et al. [133] realized a transparent Zn-based sol mixed with 3-glycidoxypropyltrimethoxysilane (GPTMS), a hybrid sol–gel precursor, to produce antimicrobial zinc-containing silica coatings on cotton fabrics. The antibacterial activity of this finishing was tested using potential pathogenic bacteria, namely *Staphylococcus aureus* and *Klebsiella pneumonia*. The obtained results were promising, showing that the sol–gel synthesized coatings based on nano-Zn acetate without and with GPTMS have high bactericidal and bacteriostatic activities. In another work, wool and silk were treated with a colloidal silver nano sol by the pad–dry–cure method and the treated materials exhibited superior antimicrobial activity [134]. Moreover, synthetic textiles were treated with nanoparticles [135]. For instance, Mahltig et al. reported a polyamide fabric with an antimicrobial-modified SiO_2_ sol containing a silver component. The resistance of the coated polyamide against *Escherichia coli* was investigated, exhibiting significant antimicrobial effects even after 40 washing cycles [136]. Apart from silver, other metals were also employed to fabricate antimicrobial textiles based on nanoparticles. For instance, Berendjchi et al. prepared sol–gel silica loaded with copper nanoparticles to manufacture an antibacterial cotton fabric. Tetraethyl orthosilicate was used as a hydrolyzed precursor and condensed in water to obtain the colloidal silica nanoparticles at room temperature. The antibacterial activity against *E. coli* and *Staphylococcus aureus* was investigated. Results showed that the percentage of bacteria growth was reduced by about 70% for *E. coli* and 90% for *S. aureus* bacteria [137]. Moreover, a recent review paper [138] reported using a biopolymer, namely chitosan, with zinc oxide, titanium oxide, and silver nanoparticles in antimicrobial textile treatment.

### 6.3. Biopolymers as Antimicrobial Agents

Researchers have extensively investigated the potential application of various natural molecules as antimicrobial agents. Apart from being safe, non-toxic, skin- and environment-friendly, some natural components have antioxidant properties and antimicrobial activity. For this reason, in the last decades, these bioactive substances have been studied. They are expected to gradually replace the conventional synthetic formulations soon as they are favorable in cost, performance, and environmental concerns. The use of chitosan to confer antimicrobial properties to textile materials dates back to 1974. It has been the focus of several scientific research works over the last few decades. This biopolymer is the second most abundant globally, following cellulose, meaning an easy availability at a low cost. Chitosan is a polysaccharide derived from the alkaline deacetylation of chitin, which can be found mainly in exoskeletal shells of crustaceans and the cell walls of fungi [139]. However, several studies have confirmed that some factors can negatively influence the antibacterial activity of the biopolymer, namely the degree of polymerization, which should be at least seven, and the pH of the aqueous medium, which needs to be under five, as above it becomes water-insoluble. Water-soluble chitosan derivatives can be obtained by the quaternization of its amino groups, which introduces permanent positive charges in the polymer chains, resulting in a cationic polyelectrolyte whose solubility is independent of the pH value [140]. For this purpose, several research studies were conducted, and modified chitosan was applied as coatings for wool and other fabrics [141]. Another issue related to using this biopolymer to functionalize textile surfaces is the weak bonds that chitosan can establish with fibers, resulting in the low durability of the treatment. Various cross-linking agents, such as polycarboxylic acids, were used on cellulosic-based fibers to address this drawback. In particular, 1,2,3,4- butane tetracarboxylic acid (BTCA) and citric acid were employed as greener cross-linking agents between chitosan and cotton, enhancing the antimicrobial durability of the finishing [142]. Moreover, the UV-radiation method was used for imparting durable antimicrobial properties to fabrics, mainly due to the advantages of this technology, such as energy savings, low environmental impact, high and straightforward treatment speed [143]. For instance, Ferrero and Periolatto [143] used UV light with a suitable photoinitiator to apply chitosan to natural and synthetic textiles. Antimicrobial tests showed that chitosan UV-curing yielded high antimicrobial properties on the selected fabrics. Moreover, cyclodextrins were used in the textile field as a green approach to fight the drawback related to bacteria growth. These molecules are cyclic oligosaccharides, consisting of glucose units linked by α-1,4-glycosidic bonds to form a truncated cone, which affords a hydrophobic space, from the inside and a hydrophilic surface, on the outside because of the peculiar arrangement of the hydroxyl groups. These compounds can be obtained by the enzymatic degradation of starch in potatoes, corn, rice, and other food items. The importance of CDs is mainly attributable to their ability to host certain compounds, that are guest molecules, inside their hydrophobic cavities by forming host-guest complexes [144]. This inclusion exerts a positive influence on the properties of the guest molecules, such as improved solubility, stability, volatility and sublimation, control of the release of active ingredients. The strength of the host-guest complex depends on several factors, such as the guest molecule size, interactions, release of water molecules. The biocompatibility and biodegradation of CDs encouraged their wide use in several fields, including the textile industry. In regard to the latter, many studies suggest that CD fixation onto cotton fabric does not affect the hydrophilic property of the material, and the immobilized cavity of CD does not lose its complexing power to form inclusion complexes with other molecules [145]. According to their chemical bonds, there are three types of CDs, namely α-CD, β-CD, and γ-CD, which include six, seven, and eight α-1,4-glycosidic bonds, respectively. Among them, β-CD has shown massive potential in cellulosic fabric modifications because of the strength and longevity of host-guest complexes, even if its solubility is the lowest of all the CDs types. This drawback can be overcome through several chemical modification methods of β-CD at the –OH groups on the exterior rims. The first reactive cyclodextrin derivative synthesis, namely monochlorotriazinyl-β-cyclodextrin (MCT-β-CD), dates back to 1996 [117]. In one research study, the optimal reaction conditions for grafting of β-cyclodextrin to cellulose fabrics were found to be MCT-β-CD 60−100 g/L, catalyst Na_2_CO_3_ 50−60 g/L, the reaction temperature of 150−160 °C and the reaction time 5−8 min. According to these findings, the MCT-β-CD grafted cellulose retained more than 70% antibacterial abilities even after washing ten cycles [117]. The pre-modification of cellulose-containing fabrics can achieve the antimicrobial activity of textile materials with β-CD or its derivatives, followed by post-treatment with antimicrobial agents [146] or by the pre-loading of an antimicrobial agent into the β-CD cavities and the consequent treatment of the material with the obtained complex [147]. Regarding the latter, a variety of antimicrobial agents were employed as guest molecules, such as silver ions, triclosan, octenidine dihydrochloride, certain antibiotics, AgNPs, and as bioactive agents such as thymol. Recently, plants received interest as a significant source of natural antimicrobials since a wide range of bioactive substances can be extracted from bark, leaves, roots, and flowers. They generally consist of coloring materials, phenolic compounds, quinones, flavonoids, tannins, polysaccharides, and essential oils [148]. These bioactive agents can be applied to textiles by using different techniques, such as direct application, cross-linking, microencapsulation, incorporation in β-CD, exhaustion method, as well as the pre-treatment of substrate surface using plasma, UV-radiation, or enzymatic treatment [149]. Several research studies on cotton fabrics demonstrated that aloe vera treatment improved their antibacterial properties.

### 6.4. Plant Extracts Used for Imparting Antimicrobial Activity to Fabrics

Aloe vera leaves possess nearly 200 active ingredients, including 75 nutrients, 20 minerals, 18 amino acids, and 12 vitamins. In one research work, cotton was finished with aloe vera gel using carboxylic acid as a cross-linker and revealed remarkable antibacterial activity against Gram-negative, Gram-positive organisms, and fungal pathogens [150]. In further research, cellulosic fabrics were finished with a combination of aloe vera and neem, showing higher durability to wash compared to those treated individually [151]. Aloe vera gel and citric acid were used to obtain an environmentally friendly, natural antimicrobial finish on the cellulosic fabric. Antibacterial tests showed that the coating provided 89% efficacy against Gram-negative, 20% against Gram-positive, and 82% against fungal pathogens [142]. In the last years, experimental studies involving tulsi leaves were conducted mainly on cotton fabrics. The main components of tulsi leaves are eugenol (70%), methyl eugenol (20%), carvacrol (3%). In one research study, the cellulosic fiber was treated with methanolic extracts of tulsi leaves by direct method and exhibited an excellent antibacterial activity [152]. In another study, cotton fabrics treated with tulsi leaves extracted via microencapsulation or resin cross-linking showed higher antibacterial activity than those treated via a direct method [153]. Turmeric (*Curcuma longa* L.) and Indian saffron (curcumin) were applied to woolen fabrics using dyeing and functional finishing process. The antibacterial property of treated textiles against *E. coli* and *S. aureus* microorganisms was investigated using the AATCC 100-1999 method. Results showed that wool fabrics treated with castor saffron provided antimicrobial activity against *S. aureus* (85% against bacteria) and *E. coli* (90% against bacteria). The antibacterial activity increased with higher dye concentration. This study investigated the washing fastness, showing that antimicrobial property decreased after 30 washing cycles (in the range of 30–45%) [154]. Moreover, cotton-knitted fabrics were treated with turmeric (*Curcuma tinctoria* Guibourt) extract with the help of various mordants using the impregnation method. The experimental results demonstrated a reduction in the range of 96.6–99.0% against microorganisms [155]. Several research works involve Henna as an antimicrobial agent. In one study, this bioactive agent was combined with chitosan in wool fabrics, and results showed an antimicrobial activity of the treated fabric [156]. In another study, cotton items were dyed with Henna (*Lawsonia inermis*) with various mordants using the impregnation method. The results demonstrated that the treated fabrics reduced the bacterial growth against *S. aureus* by 96.6% and against *E. coli* by 96.4% [155]. Capsaicin (8-methyl-N-vanillyl-6-nonenamide) is the active component of *Capsicum* plants (chili peppers), grown as food and for medicinal purposes since ancient times. It is responsible for the pungency of their fruit. This nature-derived agent was used as antimicrobial finishing for wool and cotton fabrics using the sol–gel technique. The antibacterial properties of treated textiles against *E. coli* bacteria were investigated. Capsaicin-coated textiles showed more significant antibacterial activity than the fabrics treated with the silica sol–gel method without using this bioactive molecule. Furthermore, results showed that, even if some reductions in antibacterial activity occurred after laundering, capsaicin remained in the structure of the fibers [157]. Green Tea [*Camellia sinensis* (L.) Kuntze] extract, together with citric acid as a cross-linker, was used to confer antibacterial features to cotton fabrics using a pad–dry–cure method with good antimicrobial properties of treated items [158]. Moreover, wool textiles were dyed with green tea extract with the help of aluminum sulfate, and AATCC 100-1993 method was used to test the antibacterial activity of treated fabrics. The results showed that finishing brought about an 80–99.3% bacterial reduction for *Pseudomonas aeruginosa*, 85–99.3% for *Escherichia coli*, and 90–100% for *Staphylococcus aureus* [159]. In addition, the antimicrobial activity of Licorice or Liquorice (*Glycyrrhiza glabra*) was studied in different regions of the world over the last decade. In an experimental study, *Glycyrrhiza glabra* roots were used to confer antibacterial properties to woolen fabrics. Two different types of bacteria, namely *Staphylococcus aureus* and *Escherichia coli*, were used in the tests. According to the results, *Glycyrrhiza glabra* roots exhibited good antimicrobial properties against *Staphylococcus aureus* and *Escherichia coli* bacteria species. Light and wash fastness tests of dyed fabrics were also carried out, showing the fastness properties required for the coloring of woolen fabrics [160].

### 6.5. Antimicrobial Properties of Essential Oils

In recent times, the application of Essential oils for antimicrobial purposes on textiles has increased, due to their high efficiency, even if the actual action against microbes is unclear. Consisting of various aromatic compounds, they can protect a broad spectrum of microbes. Essential oils, such as neem, clove, lavender, rosemary, and cinnamon, were applied to cellulosic fabrics by different techniques to impart durable antimicrobial coatings [161].

Although conventional commercial antimicrobial agents can protect textile materials from microbial attack, even after repeated washing cycles and ironing conditions, the majority of them have a negative impact both on the environment and on human health. The ecological and economic restrictions urged researchers to undertake more extensive work to find renewable suitable antimicrobial resources in the last decades. Among different approaches, nanotechnology and the incorporation of biologically active components have recently gained attention. However, some of their significant limitations are their poor durability and fastness properties. Therefore, the future scope of applications of these bioactive substances still requires further research to improve the incorporation method to retain antimicrobial agents on textiles for a longer time.

## 7. Conclusions

Some conclusions drawn from this comprehensive review on eco-sustainable solutions are listed as follows: This review is focused on antibacterial and antifouling agents for concrete, Cultural Heritage, filtration membrane technology, and marine and healthcare applications. (1) It is still challenging to achieve a versatile protocol to fabricate adequate and long-lasting antifouling coatings regarding the desired requirements. Producing new polymers may still be essential to enhancing antifouling performance, focusing mainly on long-term durability in static and dynamic environments. Besides surface chemistry and structure, novel surface modification and techniques need to be developed to translate these coatings into industrial applications successfully. (2) The evolution of nanotechnology applications in concrete development is almost innovative. When nanometric additives were mixed with cement and concrete, the hydration accelerated, and the rate of hydration increased with several extra functionalities. Nanotechnology can enhance concrete performance and produce sustainable and advanced cement-based composites with unique mechanical and electrical features. (3) “Green conservation” is innovation and eco-sustainability in the Cultural Heritage field. The chemical treatment for biocidal and antifouling procedures is currently the most versatile way to produce innovative solutions. Tunable solvent properties and antimicrobial and surface activity are some of the most exciting features of biotechnology and nanotechnology. This technology access to advanced materials can produce new formulations of antifouling and antimicrobial surface coatings, developed as gel materials and other forms. Furthermore, research on the design of biodegradable materials has progressed considerably, leading to the development of green characteristics, such as low volatility and recyclability. (4) Nanoparticles can be used to hydrophilize the membrane surface to enhance the anti-fouling performance [162]. However, a suitable surface coating design should be developed rather than nanoparticles blend or entrapped membranes to guarantee the best exposure and interaction with foulants. Metal–Organic Frameworks are promising nanomaterials that can be hydrophilized and functionalized with metallic nanoparticles, enhancing catalytic and hydrophilic properties. The design of innovative nanomaterials and classes of synthetic polymers allow the research and development in the field of high-performance, multi-functional, anti-fouling membranes. Innovation in characterization procedures is fundamental for membrane processes to obtain the proper insight into materials’ behavior at the nano-scale. The demand for water causes increasing cost and energy consumption, and decreasing natural reserves of conventional fuels serve as the driving force for the research and innovation in membrane separation processes. (5) Anti-fouling technologies for marine applications are of interest due to the economic and environmental benefits because coatings are non-toxic, nonbiocide-release. (6) Moreover, new technologies can produce durable and suitable curing procedures for marine coatings, including the additional advantage of shorter treatment times, cheaper maintenance costs, therefore, a significantly lower environmental impact. To increase the lifespan and enhance the anti-fouling functionality versus the unavoidable occurrence of abrasion and wear of the coated surfaces, the most desired breakthrough may come from combining anti-fouling functionality with self-healing properties automatically self-repair the anti-fouling character. The contribution of researchers, scientists, and engineers from material science, chemical engineering, environmental engineering, and mechanical engineering can bring significant breakthroughs in developing innovative hybrid antibacterial and anti-fouling solutions.

## Figures and Tables

**Table 1:** List of the common antibacterial agents used to preserve cement structures.

Antibacterial Agents	Authors	Ref.
ZnO and MgO NPs	Singh et al.	[21]
Metal zeolites and antibacterial polymeric fibers	De Muynck et al.	[29]
Epoxy resins	Kong et al.	[30]
Quaternary ammonium compounds	Javaherdashti et al.	[31]
Halogenated complex	Qiu et al.	[32]
Metal oxide, silver, and tungsten powder	Plutino et al.	[33]
CuO, Cu_2_O, ZnO, TiO_2_, Al_2_O_3_, and Fe_3_O_4_ nanoparticles	Sikora et al.	[34]
Silver nanoparticles in commercial silica-based coating	Nam, K.Y.	[35]
ZnO, TiO_2_, SiO_2_ nanoparticles	Dyshlyuk et al.	[36]
SiO_2_–Ag nanohybrid compounds in acrylic coatings	Le et al.	[37]
Silver nanoparticles in N-SiO_2_ nanocarriers	Dominguez et al.	[38]
BiOCl_x_Br_1−x_ micro flowers	Gao et al.	[39]
TiO_2_ nanoparticles, fluorine silicon sol	Zhu et al.	[40]
TiO_2_ nanoparticles	Verdier et al.	[41]
TiO_2_ modified with carbon and nitrogen	Janus et al.	[42]
TiO_2_ and ZnO nanoparticles in addition to polyethylene glycol (PEG)	Dehkordi et al.	[43]
Fe_2_O_3_ contained in steel slag of an industrial induction furnace	Baalamurugan et al.	[44]
Fly ashes recycled by alkali activation process supported with Zn	Rodwihok et al.	[45]
Metakaolin-based geopolymer cement loaded with 5-chloro-2-(2,4-Dichlorophenoxy) phenol	Rubio-Avalos, J.C.	[46]
Metakaolin-based geopolymer cement loaded with glass waste	Dal Poggetto et al.	[47]
Zinc particles or zinc doped clay particles	Roghanian et al.	[48]
Granular activated carbon and fundamental oxygen furnace steel slag particles, copper, and cobalt as inhibitory metals	Justo-Reinoso et al.	[49]

**Table 2:** List of most common biocides used.

Commercial Product and Active Ingredient	Solvent	Spectrum of Action	Ref.
Biotin T (CTS) di-n-decyl-dimethylammoniumchloride	water	fungi, bacteria, and algae	[67]
Biotin T (CTS) 3-iodo-2-propynylbutyl carbammate	ethanol	fungi, bacteria, and algae	[68]
Rocima. 103 (CTS) di-n-decyl-dimethylammoniumchloride	water	lichens, fungi, bacteria, and algae	[67,68]
Preventol RI80 (CTS) alchyl-dimethyl-benzilammoniumchloride	water	fungi, bacteria, and algae	[67,68]

**Table 3:** Different antifouling coatings for filtration membranes.

**Class of Functional Molecule**	**Deposition Method**	**Functional** **Molecules**	**Coated Membrane**	**Antifouling** **Capabilities**	**Ref.**
Zwitterionic copolymer	Dip-coating	SBMA and MTAC	PVC-PAN-PSS	Anti-organic fouling and anti-biofouling	[76]
Zwitterionic copolymer	iCVD	PDE and 1,3-propane sultone	RO	Anti-biofouling	[77]
Zwitterionic copolymers	si-ATRP	HEMA, MPC and SBMA	RO	Anti-biofouling	[78]
Star-shaped block copolymer	Self-assembly	PS core and PEGMA, PDMAEMA, PMAA arms	PSF	Anti-oil fouling	[79]
Mussel-inspired	Dip-coating	Fluorinated polyamine on PDA layer	PES	Anti-organic fouling and fouling-release	[80]
Hybrid mussel-inspired	In situ polymerization	Polydopamine–Zn complex	PSF	Anti-fouling	[81]
Zwitterionic polymer and metal NPs	Dip-coating	TA, AgNPs and zwitterionic PEI	PES	Anti-bio fouling	[82]
Mussel-inspired and metal NPs	In situ reduction	Catechol, Ag NPs, chitosan-polyurethane	PES	Anti-bio fouling	[83]
Biopolymer and MOF	Dip-coating	Cu-BTC, chitosan	PES	Anti-bio fouling	[84]
Photocatalyst	Dead-end filtration	ZnIn_2_S_4_	PVDF	Anti-organic fouling	[85]
Biopolymer	LBL	CNC, T-CNF, PAHCl	PES	Anti-bio fouling	[86]
Biopolymer	LBL	Kraft lignin, pDAC	PES	Anti-oil fouling	[87]
Zwitterionic organosilica polymer	Sol–gel and filtration	Zwitterionic organosilica monomer	PVDF	Antifouling and anti-bioadhesion	[88]
Silica NPs and mussel-inspired	Sol–gel	TEOS, polydopamine	PVDF	Anti-oil fouling	[89]

**Table 4:** Different antifouling technologies for paints.

Antifouling Technology	Properties	Mechanism of Action	Ref.
Contact leaching coatings	Biocides are incorporated into water-insoluble matrices	Biocidal paint, dissolution of water-soluble biocides that are released gradually	[98]
Controlled depletive polymer (CDP) coatings	Biocides are incorporated in a resin-based soluble matrix	Biocidal paint, physical dissolution of the soluble matrix, and release of the biocides	[98]
Self-polishing copolymer (SPC) coatings	Biocides are incorporated in an acrylic or methacrylate polymer matrix	Biocidal paint, decomposition by hydrolysis of the matrix detaches organisms from the hull, releasing biocides	[98]
CDP and SPC mixed coatings	It combines the properties of the CDP and SPC technologies	Biocidal paint, dissolution by hydrolysis, and hydration of the matrix with control of the biocide release	[98]
Zwitterionic polymer coatings	It combines amphiphilic and zwitterionic groups on the surface of the coatings.	Non-biocidal paint, formation of a hydration layer around zwitterionic moiety	[102,103]
Silicone or fluorine-based coatings	Combination of polymers with low surface energy and modulus.	Foul-release paint, minimization of the adhesion force between the foulers and the material of which the hull surface is made	[104,105,106]
Conductive antifouling coatings	Use of negative charges on the surface of the film.	Foul-release paint, high electrostatic repulsion between the films and the foulants	[107]

## Data Availability

The data presented in this study are available on request from the corresponding author.
